# Gene Expression Analysis of Autophagy Markers in Primary and Secondary Myelofibrosis

**DOI:** 10.3390/jcm14072333

**Published:** 2025-03-28

**Authors:** Marin Medugorac, Katarina Marija Glick, Ana Livun, Marko Lucijanic, Davor Galusic, Rajko Kusec

**Affiliations:** 1 Division of Hematology, Department of Internal Medicine, University Hospital Centre Zagreb, 10000 Zagreb, Croatia; 2 Division of Molecular Diagnostics and Genetics, Department of Laboratory Diagnostics, University Hospital Dubrava, 10000 Zagreb, Croatia; 3 Department of Scientific Research and Translational Medicine, University Hospital Dubrava, 10000 Zagreb, Croatia; 4 Division of Hematology, Department of Internal Medicine, University Hospital Dubrava, 10000 Zagreb, Croatia; 5 School of Medicine, University of Zagreb, 10000 Zagreb, Croatia; 6 Division of Hematology, Department of Internal Medicine, University Hospital Centre Split, 21000 Split, Croatia; 7 School of Medicine, University of Split, 21000 Split, Croatia

**Keywords:** autophagy, Beclin-1, LC3B-II, myelofibrosis

## Abstract

**Background/Objectives**: According to previous research, the process of autophagy in myeloid neoplasms has proven to be ambivalent depending on the type and stage of the disease. The aim of our work was to investigate the mechanism of autophagy in patients with primary and secondary myelofibrosis. **Methods**: Based on the RT-PCR method, we retrospectively analyzed the expression of *Beclin-1* and *LC3B-II* in bone marrow cells of patients with primary and secondary myelofibrosis (74 participants) compared to the control group which had patients with lymphoma in a localized stage without bone marrow infiltration (11 participants). **Results**: There was no statistically significant difference in the expression of *Beclin-1* and *LC3B-II* between patients with primary and secondary myelofibrosis and control participants. Among patients with primary myelofibrosis, higher expression of *LC3B-II* was statistically significantly associated with lower DIPSS. Higher *Beclin-1* expression was statistically significantly associated with better patient survival. **Conclusions**: Our results suggest that the upregulation of autophagy genes may be associated with favorable prognosis and survival of patients with myelofibrosis.

## 1. Introduction

Autophagy is an evolutionarily conserved mechanism of degradation of senescent organelles and macromolecules, essential for maintaining cell homeostasis [1,2]. Induction of autophagy occurs in states of increased energy needs and oxidative stress [1,2]. Autophagy is a sequential process that begins with nucleation (the formation of an isolation membrane or phagophore) and ends with the fusion of autophagosome with the lysosome [1,2]. Beclin-1 and LC3B-II (membrane-bound microtubule-associated protein 1 light chain 3 beta) are the most commonly used autophagy markers [1,2]. Different types of stress stimuli, including nutrient starvation [3], hypoxia [4], oxidative stress [5], misfolded protein accumulation [6], mitochondrial [7] and DNA damage [8], invasion by pathogens [9], mechanical stress [10] and psychological stress [11], can affect activation of autophagy. The phosphatidylinositol 3-kinase (PI3K)/protein kinase B (AKT)/mammalian target of rapamycin (mTOR) is a key intracellular signaling pathway regulating the activation of autophagy [12]. Under normal cell conditions, a basal level of autophagy is necessary to maintain homeostasis and eliminate old or damaged proteins and organelles [13,14]. Moreover, autophagy is required to maintain unique properties of hematopoietic stem cells and balance processes of their self-renewal and differentiation [15,16]. However, regarding cancer biology, autophagy may play an ambiguous role, both promoting and suppressing processes that may aid in cancer survival or death [17].

Chronic myeloproliferative neoplasms (MPNs) are malignant diseases of hematopoietic stem cells, exerting indolent clinical behavior considering the risks of clonal progression into more aggressive myeloid malignancies [18]. Among three traditional BCR::ABL negative MPN entities, polycythemia vera (PV), essential thrombocythemia (ET), and primary myelofibrosis (PMF), PMF exerts the highest tendency for progression and death [19]. It is characterized by neoplastic proliferation of megakaryocytes and granulocytes, fibrosis of the bone marrow, and extramedullary hematopoiesis in an enlarged spleen. In most patients, somatic mutations in the genes *JAK2^V617F^*, *CALR* or *MPL* are detected [20]. Secondary myelofibrosis (SMF) results from transformation of polycythemia vera (post-PV SMF), or essential thrombocythemia (post-ET SMF), and the clinical and laboratory characteristics of the disease are overall similar to PMF although some differences in specific risks may exist [21,22]. Previous research has established that the role of autophagy in the development of myeloid neoplasms is ambivalent, depending on the type and stage of disease and the use of certain drugs [23,24]. The aim of our research was to investigate autophagy by measuring the expression of the *Beclin-1* and *LC3B-II* genes in the bone marrow cells of patients suffering from primary and secondary myelofibrosis and examine the correlation with clinical and hematological parameters.

## 2. Materials and Methods

### 2.1. Patients and the Methodology

The study included 74 participants in the test group and 11 in the control group. The bone marrow was sampled as part of the standard diagnostic algorithm with previously obtained informed consent of the participants and the approval of the Ethics Committee. The test group included patients with PMF or post-ET and post-PV SMF evaluated at the time of diagnosis in the Hematology department of University hospital Dubrava, Zagreb in period from 2007 to 2021. Diagnoses were reassessed according to the 2022 WHO and ICC criteria [18,25]. The control group included patients suffering from lymphoma in the localized stage, sampled at the time of diagnosis, before the introduction of specific therapies. The study samples were bone marrow aspirates which were informative of bone marrow morphology, obtained at the time of bone marrow biopsy procedure. Mononuclear cells were isolated from bone marrow aspirates using Histopaque (Sigma, St. Louis, MO, USA; density 1.077 g/mL) and preserved in liquid nitrogen using DMSO until needed. RNA was isolated from bone marrow cells using the QIAamp RNA Blood Mini Kit (Quiagen, Hilden, Germany, Cat. nr. 52304). After measuring the concentration on a Qubit 4 Fluorometer (Thermo Fisher Scientific, Waltham, MA, USA), the RNA was reverse-transcribed into cDNA using High-Capacity cDNA Reverse Transcription Kit (Applied Biosystems, Foster City, CA, USA, Cat. nr. 4368814) and amplified with Brilliant II QPCR High Rox Master Mix (Alphachrom, Agilent Technologies, Santa Clara, CA, USA, Cat. nr. 600805). The relative expressions of *Beclin-1* (Thermo Fisher Scientific, TaqMan expression assay ID: Hs00186838_m1) and *LC3B-II* (Hs00797944_s1) were quantified by RT-PCR (real-time polymerase chain reaction) on an ABI Prism 7300 Sequence Detection System (Applied Biosystems). Expression was normalized by comparison with the *GUSB* (beta glucuronidase) gene (Thermo Fisher Scientific, TaqMan Assay ID: Hs99999908_m1). Relative expression (∆Ct, delta cycle threshold) was calculated as the difference between the mean Ct value of the endogenous control and the mean Ct value of the gene of interest (∆Ct = μCt*_GUSB_* − μCt*_Gene of interest_*). Relative expressions are shown as the difference in cycle number (∆Ct; higher value corresponds to higher expression). No significant difference was present regarding the date of sample acquisition in either of the tested genes (CT and delta CT values). Complete blood count was obtained using the Advia 2120i counter (Siemens Medical Solutions Diagnostics Pte Ltd., Swords, Ireland). Biochemical parameters were determined using the AU5800 analyzer (Beckman Coulter, Tokyo, Japan) and original manufacturer reagents.

### 2.2. Statistical Methods

The normality of the distribution of numerical variables was tested with the Shapiro–Wilk test. Because most numerical variables did not have a normal distribution, numerical variables were presented as median and interquartile range (IQR) and were compared between groups using Mann–Whitney *U* test and the Kruskal–Wallis ANOVA test. Two numerical variables were compared with each other using Spearman’s correlation. Categorical variables were presented as frequencies and percentages and were compared between groups using the Chi-squared (χ^2^) test. Survival analysis was based on the Kaplan–Meier method, and the log-rank test was used. ROC curve analysis was used to select the optimal threshold value of the expression of individual genes for survival analysis. *p* values < 0.05 were considered statistically significant. In Table 1 and Table 2, significant associations are denoted with * if statistical significance was present when the variable of interest was dichotomized and ** if the variable of interest was treated as a continuous variable; *p* values in tables are reported for dichotomized context and may be denoted with ** even if above 0.05, hence implying there was a significant association in alternative analysis. No formal adjustments of the *p* values for multiple presented comparisons were made. The statistical program MedCalc version 20.110 (MedCalc Software Ltd., Ostend, Belgium) was used for all analyses.

Considering required sample size, we conducted a pre-study power analysis based on an assumption that a 10-fold increase in a specific gene relative expression would represent meaningful difference, which would correspond to ∆Ct difference of 3.32. Assuming a difference in ∆Ct value of 3.32, standard deviations of 3 in both groups, ratio of patients in subgroups of 7:1, type I error of 0.05, and 80% statistical power, it would be required to include a total of 61 patients (53 and 8 in specific subgroups) to detect statistically significant difference.

## 3. Results

**Figure 1:**
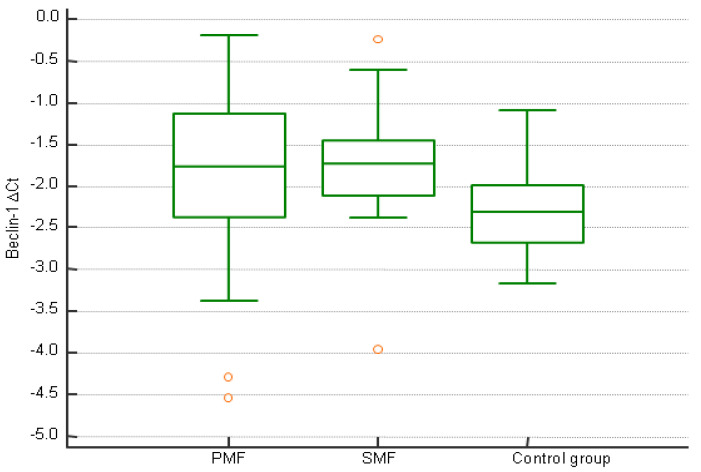
*Beclin-1* expression in patients with PMF, patients with SMF, and control group.

Out of 74 participants in the test group, there were a total of 42 (56.8%) men and 32 (43.2%) women. The median age was 67 years (IQR 58–75). The majority of patients were *JAK2^V617F^*-mutated (42 (67.2%)). A total of 56 (75.7%) patients had PMF, 10 (13.5%) post-PV SMF, and 8 (10.8%) post-ET SMF. According to DIPSS (dynamic international prognostic scoring system), 9 (18.8%) PMF patients were at low risk, 25 (52.1%) at intermediate-1, 9 (18.8%) at intermediate-2, and 5 (10.4%) at high risk. According to Mysec-PM (prognostic model for secondary myelofibrosis), 1 (8.3%) SMF patient was at low risk, 4 (33.3%) were at intermediate-1, 4 (33.3%) at intermediate-2, and 3 (25%) at high risk. Median follow-up was 40 months. The 5-year survival rate was 44%. Out of 11 control participants, there were a total of 9 (81.8%) men and 2 (18.2%) women. The median age was 46 years (IQR 41–57). There was no statistically significant difference in *Beclin-1* expression between patients with PMF, patients with SMF, and control group (*p* = 0.112, Figure 1).

When *Beclin-1* expression in the test group was stratified on the median, higher expression was statistically significantly associated with lower absolute number of basophils and lymphocytes, lower percentage of circulatory blasts, lower RDW (red cell distribution width), higher platelet count, lower MPV (mean platelet volume), lower LDH (lactate dehydrogenase), and lower CRP (C-reactive protein) (*p* < 0.05 for all comparisons, Table 1). There was no statistically significant difference in *Beclin-1* expression depending on age, sex, origin of myelofibrosis, grade of bone marrow fibrosis, JAK2 mutational status, nor DIPSS disease risk in PMF and Mysec-PM disease risk in SMF patients (*p* > 0.05 for all comparisons, Table 1).

**Figure 2:**
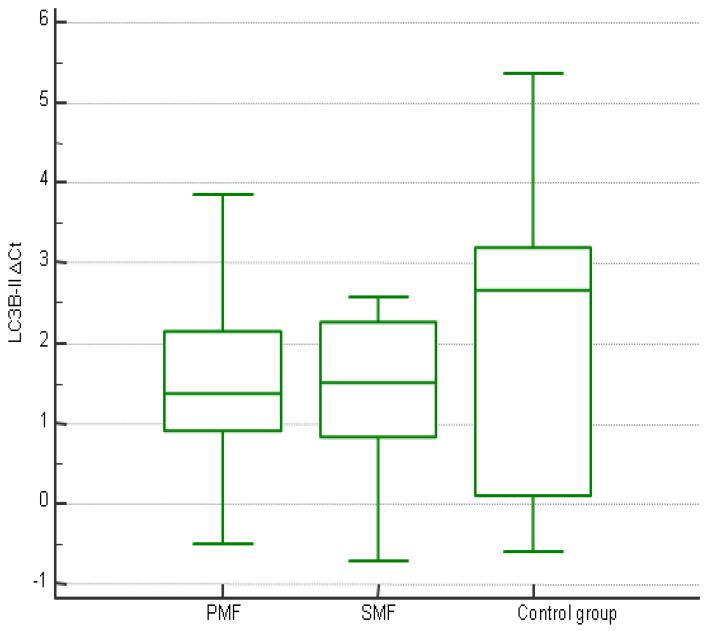
*LC3B-II* expression in patients with PMF, patients with SMF, and control subjects.

There was no statistically significant difference in the expression of *LC3B-II* between patients with PMF, patients with SMF, and the control group (*p* = 0.561, Figure 2).

**Figure 3:**
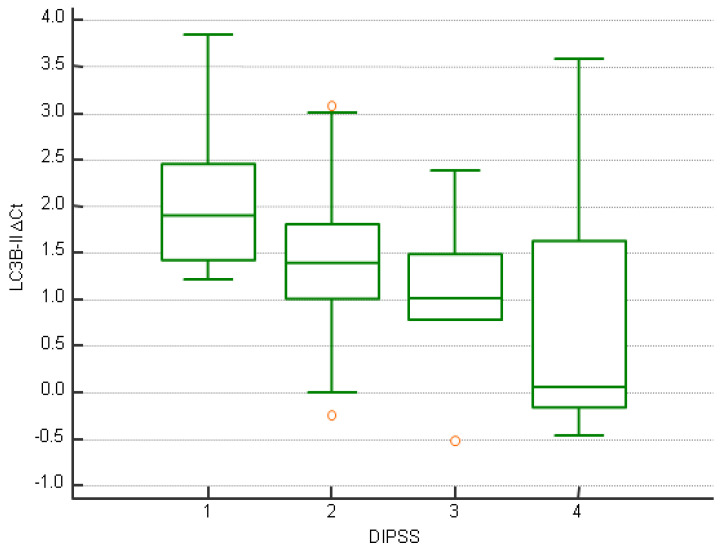
*LC3B-II* expression depending on DIPSS risk category in PMF patients (1 = low risk, 2 = intermediate-1 risk, 3 = intermediate-2 risk, 4 = high risk).

After stratification on the median, higher *LC3B-II* expression was statistically significantly associated with lower absolute monocyte and lymphocyte counts, lower LDH, lower serum iron level, lower transferrin saturation (TSAT), and lower ferritin concentration (*p* < 0.05 for all comparisons, Table 2). When it was analyzed as a continuous variable, higher *LC3B-II* expression was statistically significantly associated with lower DIPSS disease risk in PMF patients (Figure 3 and Table 2).

There was no statistically significant difference in *LC3B-II* expression depending on age, sex, origin of myelofibrosis, grade of bone marrow fibrosis, JAK2 mutational status, or Mysec-PM disease risk in SMF patients (*p* > 0.05 for all comparisons).

**Figure 4:**
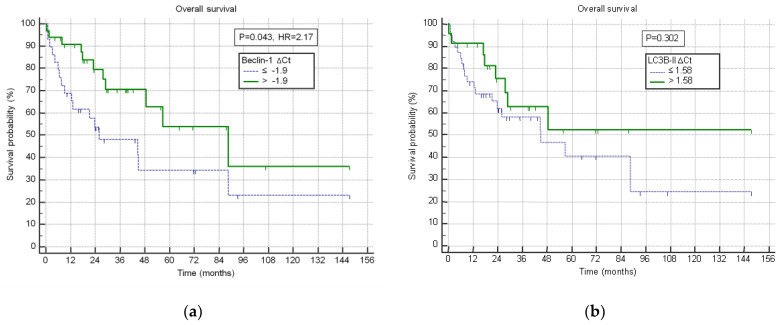
(**a**) Correlation of *Beclin-1* expression with overall survival; (**b**) correlation of *LC3B-II* expression with overall survival.

The optimal threshold values of the tested genes for survival analysis were determined by ROC curve analysis. Higher *Beclin-1* expression (∆Ct > −1.9) was statistically significantly associated with better patient survival (*p* = 0.043, Figure 4a). *LC3B-II* expression divided by optimal threshold value of ∆Ct (>1.58) was not statistically significantly associated with patient survival (*p* = 0.303, Figure 4b), although the similar trend of favorable survival was observed.

## 4. Discussion

Our research did not establish a statistically significant difference in the expression of *Beclin-1* and *LC3B-II* in bone marrow cells of patients with primary and secondary myelofibrosis compared to the control group. When *LC3B-II* expression was analyzed as a continuous variable among patients with primary myelofibrosis, higher expression was statistically significantly associated with lower DIPSS risk. As another significant result, we highlight that higher expression of *Beclin-1* was statistically significantly associated with better patient survival. To the best of our knowledge, this is the first time that expression of *Beclin-1* and *LC3B-II* was analyzed by RT-PCR on mononuclear bone marrow cells of patients with primary and secondary myelofibrosis.

In a previously published work (Shi, Guanfang et al.), the expression of *Beclin-1* and *LC3B-II* was quantified by RT-PCR and Western blot method on mononuclear cells from peripheral blood, and the results showed a lower expression of *Beclin-1* and *LC3B-II* in patients with myelofibrosis compared to healthy subjects and patients with polycythemia vera and essential thrombocythemia [26], thus implying downregulation of the process of autophagy associated with the development of bone marrow fibrosis. As emphasized in the introduction, the process of autophagy is ambivalent in the etiopathogenesis of myeloid neoplasms, depending on the type of the disease and the use of certain drugs. In silico studies on patients suffering from myelodysplastic syndrome, acute myeloid leukemia, and chronic myeloproliferative neoplasms confirmed that the expression of genes involved in the mechanism of autophagy is reduced in the blasts of the patients compared to the granulocytes of healthy subjects [23]. The mentioned genes are located in the chromosome regions lost by deletion (CDRs, commonly deleted regions) [23]. Taking into account the fact that mitochondria are the main source of ROS (reactive oxygen species), dysfunctional mitophagy results in increased oxidative stress and DNA damage [23]. It is known that many drugs used in the treatment of patients with myeloid neoplasms lead to induction of autophagy. The difference is that some result in cytoreductive and the other in cytoprotective autophagy. For example, ATRA (all-trans retinoic acid) and ATO (arsenic trioxide), which form the backbone of the treatment of patients with acute promyelocytic leukemia, result in autophagy-induced proteolysis of the fusion oncoprotein PML-RARA (promyelocytic leukemia/retinoic acid receptor alpha), meaning they induce cytoreductive autophagy [24]. Contrary to the above, it was recently reported that ruxolitinib may lead to the induction of cytoprotective autophagy, and the concomitant use of autophagy inhibitors may increase the sensitivity of *JAK2^V617F^*-positive cells to ruxolitinib [27].

Autophagy is an essential process for the development and maintenance of healthy hematopoietic stem cells and becomes especially important for normal hematopoiesis under conditions of metabolic stress [16], such as the high inflammatory burden present in patients with myelofibrosis. This housekeeping role of the autophagy mechanisms is one of the limitations in developing specific therapies aimed directly at autophagy processes. In addition, discrepancies exist in the specific roles played by autophagy inside cancer cells, potentially acting both as a tumor suppressor and promoter [28]. Autophagy may provide an alternative mechanism of cell death in cancer cells that have escaped apoptosis, and its inhibition may be effective especially in BRAF-mutated cancer cells [29]. There are several drug classes actively targeting autophagy pathways in cancer that are being developed. These include PI3K inhibitors that can block autophagosome formation [30]. Chloroquine, hydroxychloroquine, and other lysosome-tropic agents can block lysosome acidification, in turn resulting in disruption in autophagosome degradation [31]. A similar result is achieved by inhibitors of autophagosome and lysosome fusion [32]. Delivery of antitumor drugs using nanoparticles was also shown to enhance autophagy [33]. Other mechanisms such as microRNAs inhibiting the expression of key autophagy components [34] and tumor vaccines based on autophagy targets have also been investigated [35]. Although the preclinical data suggest that autophagy inhibition may mitigate development of myelofibrosis in mice [36], to the best of our knowledge, there are no active ongoing trials of autophagy inhibitors in patients with myelofibrosis at the moment. It should be noted that our current results imply that the upregulation of autophagy might be beneficial in the context of myelofibrosis, warranting special considerations in design and the implementation of such treatment approaches.

Several more favorable clinical myelofibrosis features were significantly associated with the upregulation of *Beclin-1* and *LC3B-II* in the current study. These include a lower absolute number of basophils and lymphocytes, lower percentage of circulatory blasts, lower RDW, higher platelet count, lower MPV, lower LDH and lower CRP for *Beclin-1,* lower absolute monocyte and lymphocyte counts, lower LDH, lower serum iron level, lower TSAT, lower ferritin concentration, and lower DIPSS risk score for *LC3B-II*. It seems that higher expression of autophagy genes is associated with a less proliferative disease phenotype, as judged by lower counts of leukocyte subtypes and lower LDH. Both higher basophil [37] and monocyte counts [38] have been associated with unfavorable survival in myelofibrosis patients, and the same was reported for higher ferritin [39], CRP [40] and LDH [41], RDW [42], and lower platelet count [43]. Thus, the tendency of improved long-term outcomes with higher expression of autophagy genes may be mediated by more favorable disease features. Also, the process of autophagy may play an important role outside of the context of diseased stem cells and may reflect on the biology of general metabolism [44], thrombosis [45], and cardiovascular health [46,47], which are important for long-term cardiovascular morbidity.

Limitations of our work are the retrospective study design, small sample size limiting the statistical power of analyses, as well as inability to investigate the contribution of exposure to different therapies over time that may have affected autophagy during the follow-up period. Since the study samples were bone marrow aspirates, some degree of dilution by peripheral blood could not be avoided due to technical limitations of the process and biological characteristics of the disease. We had no access to information on immature granulocyte count, besides the proportion of circulatory blasts. Multiple statistical comparisons are presented which may result in an inflated type I error rate. Due to the exploratory nature of the study, we did not attempt to control for this phenomenon, and no specific corrections were used for interpretation of obtained *p* values (to avoid increase in the type II error rates that may lead to further loss of statistical power). Thus, the current findings should be interpreted with “informal adjustments” by the readers themselves, without applying formal corrections to the raw findings, recognizing that a certain percentage of significant findings may occur by chance [48].

Considering future directions, it should be noted that autophagy inhibition that may suppress tumor clone have uncertain benefits regarding long-term effects in patients with myelofibrosis, since markers of autophagy upregulation seem to be associated with longer survival in these patients. Drugs such as metformin, which is known to initiate processes of autophagy [49], were recently reported to be potentially beneficial in preventing development of MPN [50]; however, they may not be able to revert the established bone marrow fibrosis [51]. Since long-term survival of patients with MPNs is determined by the occurrence of thrombotic events, the role of autophagy in the development and prevention of atherosclerosis and cardiovascular morbidity may also be important [52]. Also, any drugs aimed at inhibition of autophagy in malignant stem cell clones may also inadvertently affect this process in vascular tissues as well. The risk/benefit ratio of such an approach would probably depend on the aggressiveness of the malignant clone and the degree of cardiovascular burden, and would surely need to be tested in randomized controlled trials.

## 5. Conclusions

The results of our research suggest that the upregulation of autophagy genes may be associated with favorable prognosis and survival of patients with myelofibrosis.

## Figures and Tables

**Table 1:** Clinical characteristics of patients with myelofibrosis stratified according to *Beclin-1* expression divided on the median (higher ∆Ct*_Beclin-1_* = higher expression).

	∆Ct*_Beclin-1_* ≥ −1.76	∆Ct*_Beclin-1_* < −1.76	*p*
**Age (years)**	66 IQR (58–77)	67 IQR (58–73.25)	0.659
**Sex**			
Male	22/37 (59.5%)	20/37 (54.1%)	
Female	15/37 (40.5%)	17/37 (45.9%)	0.639
**Origin of myelofibrosis**			
PMF	27/37 (73%)	29/37 (78.4%)	
Post-PV SMF	5/37 (13.5%)	5/37 (13.5%)	
Post-ET SMF	5/37 (13.5%)	3/37 (8.1%)	0.751
**Grade of fibrosis of bone marrow**			
0-I	13/29 (44.8%)	12/33 (36.4%)	
II-III	16/29 (55.2%)	21/33 (63.6%)	0.498
**DIPSS (PMF)**			0.176
Low	6/21 (28.6%)	3/27 (11.1%)	
Intermediate-1	12/21 (57.1%)	13/27 (48.1%)	
Intermediate-2	2/21 (9.5%)	7/27 (25.9%)	
High	1/21 (4.8%)	4/27 (14.8%)	
**Mysec-PM (SMF)**			0.771
Low	1/8 (12.5%)	0/4 (0%)	
Intermediate-1	3/8 (37.5%)	1/4 (25%)	
Intermediate-2	2/8 (25%)	2/4 (50%)	
High	2/8 (25%)	1/4 (25%)	
**JAK2^V617F^-positive**	22/29 (75.9%)	19/32 (59.4%)	0.171
**WBC (×10^9^/L; n.v. 3.4–9.7)**	11.1 IQR (6.9–15.8)	11.5 IQR (8.3–16)	0.530
**Monocytes (×10^9^/L; n.v. 0.12–0.84)**	0.5 IQR (0.3–0.6)	0.5 IQR (0.35–0.85)	0.291
**Basophils (×10^9^/L; n.v. 0–0.06)**	0.1 IQR (0.07–0.17)	0.2 IQR (0.1–0.3)	0.032 *^,^**
**Lymphocytes (×10^9^/L; n.v. 1.19–3.35)**	1.3 IQR (1–1.7)	1.8 IQR (1.45–2.75)	0.012 *^,^**
**Circulatory blasts (%, n.v. <1)**	0 IQR (0–1)	2 IQR (0–5)	0.013 * ^,^**
**Hemoglobin (g/L, n.v. 119–157)**	125 IQR (108–142)	111 IQR (97–122)	0.093
**RDW (%, n.v. 9.0–15.0)**	18.5 IQR (15.5–20.8)	19.6 IQR (17.78–21.7)	0.050 *
**Platelets (×10^9^/L; n.v. 158–424)**	574 IQR (297–758)	342 IQR (181–550)	0.021 *
**MPV (fL; n.v. 6.8–10.4)**	8.8 IQR (8.1–9.7)	9.5 IQR (8.75–10.8)	0.024 *^,^**
**LDH (U/L; n.v. <241)**	359 IQR (234–485)	546.5 IQR (342.75–887.25)	0.004 *^,^**
**CRP (mg/L; n.v. <5)**	2.6 IQR (1.2–3.6)	4.3 IQR (2.18–13.4)	0.032 *

* statistically significant at level *p* < 0.05, *Beclin-1* expression stratified on the median. ** statistically significant at level *p* < 0.05, *Beclin-1* expression analyzed as continuous variable. Abbreviations: ∆Ct—delta cycle threshold, IQR—interquartile range, PMF—primary myelofibrosis, post-PV SMF—post-polycythemia vera secondary myelofibrosis, post-ET SMF—post-essential thrombocythemia secondary myelofibrosis, DIPSS—dynamic international prognostic scoring system, Mysec-PM—prognostic model for secondary myelofibrosis, JAK2—Janus kinase 2, WBC—white blood cell count, n.v.—normal value, RDW—red cell distribution width, MPV—mean platelet volume, LDH—lactate dehydrogenase, CRP—C-reactive protein.

**Table 2:** Clinical characteristics of patients with myelofibrosis stratified according to *LC3B-II* expression divided on the median (higher ∆Ct*_LC3B-II_* = higher expression).

	∆Ct*_LC3B-II_* ≥ 1.43	∆Ct*_LC3B-II_* < 1.43	*p*
**Age (years)**	64 IQR (58–77)	68 IQR (59.5–74.25)	0.842
**Sex**			
Male	20/37 (54.1%)	22/37 (59.5%)	
Female	17/37 (45.9%)	15/37 (40.5%)	0.639
**Origin of myelofibrosis**			
PMF	27/37 (73%)	29/37 (78.4%)	
Post-PV SMF	7/37 (18.9%)	3/37 (8.1%)	
Post-ET SMF	3/37 (8.1%)	5/37 (13.5%)	0.337
**Grade of fibrosis of bone marrow**			
0–I	14/30 (46.7%)	11/32 (34.4%)	
II–III	16/30 (53.3%)	21/32 (65.6%)	0.324
**DIPSS (PMF)**			0.068 **
Low	7/22 (31.8%)	2/26 (7.7%)	
Intermediate-1	12/22 (54.5%)	13/26 (50%)	
Intermediate-2	2/22 (9.1%)	7/26 (26.9%)	
High	1/22 (4.5%)	4/26 (15.4%)	
**Mysec-PM (SMF)**			0.560
Low	0/7 (0%)	1/5 (20%)	
Intermediate-1	3/7 (42.9%)	1/5 (20%)	
Intermediate-2	2/7 (28.6%)	2/5 (40%)	
High	2/7 (28.6%)	1/5 (20%)	
**JAK2^V617F^-positive**	23/30 (76.7%)	18/31 (58.1%)	0.122
**WBC (×10^9^/L; n.v. 3.4–9.7)**	10.3 IQR (7.35–15.05)	13.1 IQR (8.23–17.85)	0.130
**Monocytes (×10^9^/L; n.v. 0.12–0.84)**	0.4 IQR (0.3–0.6)	0.6 IQR (0.4–0.9)	0.021 *^,^**
**Basophils (×10^9^/L; n.v. 0–0.06)**	0.1 IQR (0.02–0.2)	0.2 IQR (0.1–0.25)	0.060 **
**Lymphocytes (×10^9^/L; n.v. 1.19–3.35)**	1.3 IQR (1–1.6)	1.8 IQR (1.5–2.8)	0.004 *^,^**
**Circulatory blasts (%, n.v. <1)**	0 IQR (0–1.33)	0 IQR (0–3.65)	0.320
**Hemoglobin (g/L, n.v. 119–157)**	121 IQR (108–132)	116 IQR (97–127)	0.223
**RDW (%, n.v. 9.0–15.0)**	18.7 IQR (16.93–20.5)	19.6 IQR (17.15–22.35)	0.207
**Platelets (×10^9^/L; n.v. 158–424)**	546.5 IQR (281.25–683.5)	351.5 IQR (179.25–570.25)	0.128
**MPV (fL; n.v. 6.8–10.4)**	9.1 IQR (8.15–9.7)	9.3 IQR (8.43–10.68)	0.336
**LDH (U/L; n.v. <241)**	375.5 IQR (252.75–556.6)	515 IQR (341.5–744.5)	0.045 *^,^**
**CRP (mg/L; n.v. <5)**	3.2 IQR (1.65–10.3)	3.5 IQR (1.3–8.55)	0.960
**Fe (μmol/L; n.v. 11–32)**	12 IQR (9.75–14)	15.9 IQR (11.9–18.4)	0.016 *^,^**
**TIBC (μmol/L; n.v. 49–72)**	55.6 IQR (48.5–58.45)	50.2 IQR (46.3–53.25)	0.092 **
**TSAT (%, n.v. >20)**	21.7 IQR (13.8–28.38)	30.2 IQR (24.18–35.85)	0.013 *^,^**
**Ferritin (μg/L; n.v. 10–120)**	83 IQR (18–208)	232.5 IQR (177.75–352.25)	0.027 *^,^**

* statistically significant at level *p* < 0.05, *LC3B-II* expression stratified on the median. ** statistically significant at level *p* < 0.05, *LC3B-II* expression analyzed as continuous variable. Abbreviations: ∆Ct—delta cycle threshold, LC3B-II—membrane-bound microtubule-associated protein 1 light chain 3 beta, IQR—interquartile range, PMF—primary myelofibrosis, post-PV SMF—post-polycythemia vera secondary myelofibrosis, post-ET SMF—post-essential thrombocythemia secondary myelofibrosis, DIPSS—dynamic international prognostic scoring system, Mysec-PM—prognostic model for secondary myelofibrosis, JAK2—Janus kinase 2, WBC—white blood cell count, n.v.—normal value, RDW—red cell distribution width, MPV—mean platelet volume, LDH—lactate dehydrogenase, CRP—C-reactive protein, TIBC—total iron binding capacity, TSAT—transferrin saturation.

## Data Availability

The raw data supporting the conclusions of this article will be made available by the authors on request.
